# Supplementary material for: Metabolomic Insights into the Phytochemical Profiles and Seasonal Shifts of Fucus serratus and F. vesiculosus Harvested in Danish Coastal Waters (Aarhus Bay)—An Untargeted High-Resolution Mass-Spectrometry Approach
Source: Mar Drugs. 2025 Oct 26;23(11):417. doi: 10.3390/md23110417 (PMC12654025; doi:10.3390/md23110417)
Supplement: Supplementary file 1 [file marinedrugs-23-00417-s001.zip › marinedrugs-3918645-supplementary.pdf]

marine drugs

MDPI

Article

Metabolomic Insights into the Phytochemical Profiles and Seasonal Shifts of *Fucus serratus* and *F. vesiculosus* Harvested in Danish Coastal Waters (Aarhus Bay)—An Untargeted High-Resolution Mass-Spectrometry Approach

Mihai Victor Curtasu [1,*], Jørgen Ulrik Graudal Levinsen [2], Annette Bruhn [2,3], Mette Olaf Nielsen [1] and Natalja P. Nørskov [2]

[1] Department of Animal and Veterinary Sciences, AU Campus Viborg, Research Centre Foulum, Aarhus University, DK-8830 Tjele, Denmark; mon@anivet.au.dk

[2] Department of Ecoscience, Aarhus University, DK-8800 Aarhus, Denmark; jugl@ecos.au.dk (J.U.G.L.); anbr@ecos.au.dk (A.B.); natalja.norskov@anivet.au.dk (N.P.N.)

[3] Centre for Circular Bioeconomy (CBIO), AU Campus Viborg, Research Centre Foulum, Aarhus University, DK-8830 Tjele, Denmark

* Correspondence: mihai.curtasu@anivet.au.dk

SUPPLEMENTARY MATERIALS

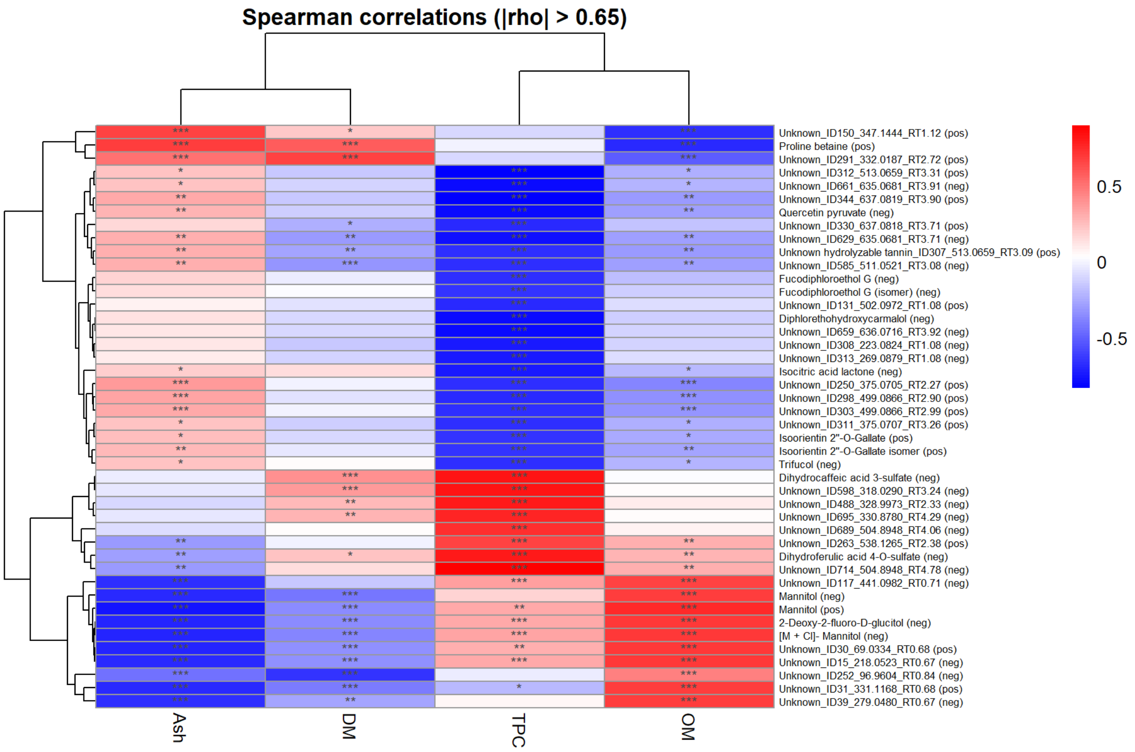

Supplementary Figure S1. Spearman correlations (|ϱ| > 0.65) between metabolite features and com-positional variables (Ash, DM, TPC, OM) in *Fucus* species. Rows and columns are hierarchically clustered. Significance after FDR adjustment: p<0.05 (*), p<0.01 (**), p<0.001 (***).

 https://doi.org/10.3390/md23110417

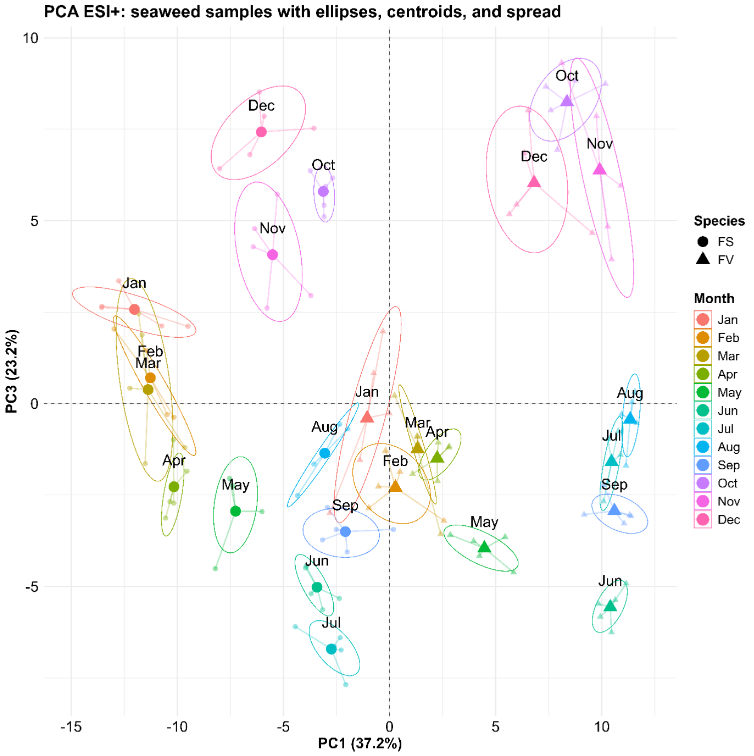

Supplementary Figure S2a. Principal Component Analysis of seasonal metabolomic shifts in *Fucus serratus* (FS) and *Fucus vesiculosus* (FV) observed in ESI+ mode. Comparison of PC1 (37.2%) and PC3 (23.2%)

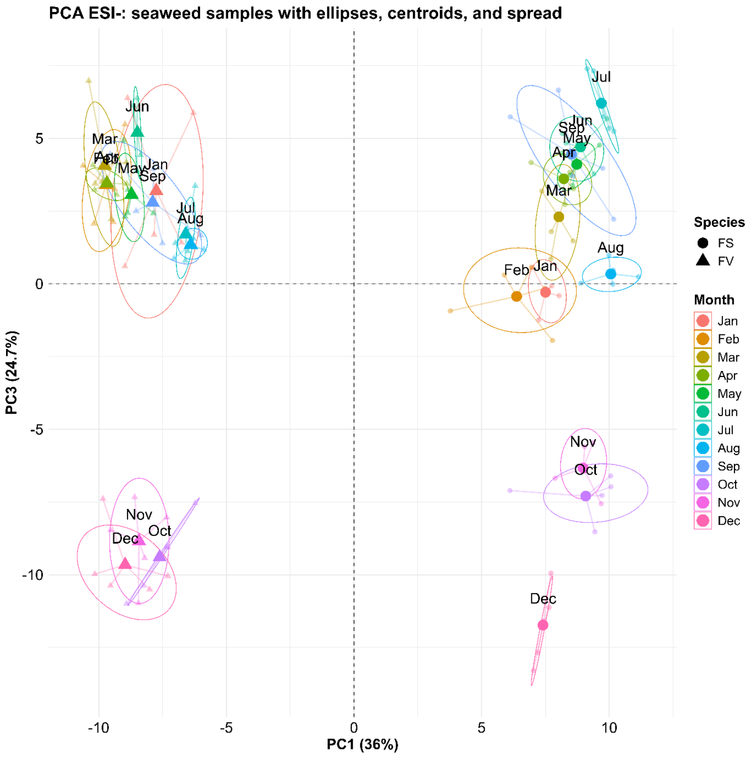

Supplementary Figure S2b. Principal Component Analysis of seasonal metabolomic shifts in *Fucus serratus* (FS) and *Fucus vesiculosus* (FV) observed in ESI- mode. Comparison of PC1 (36 %) and PC3 (24.7%)

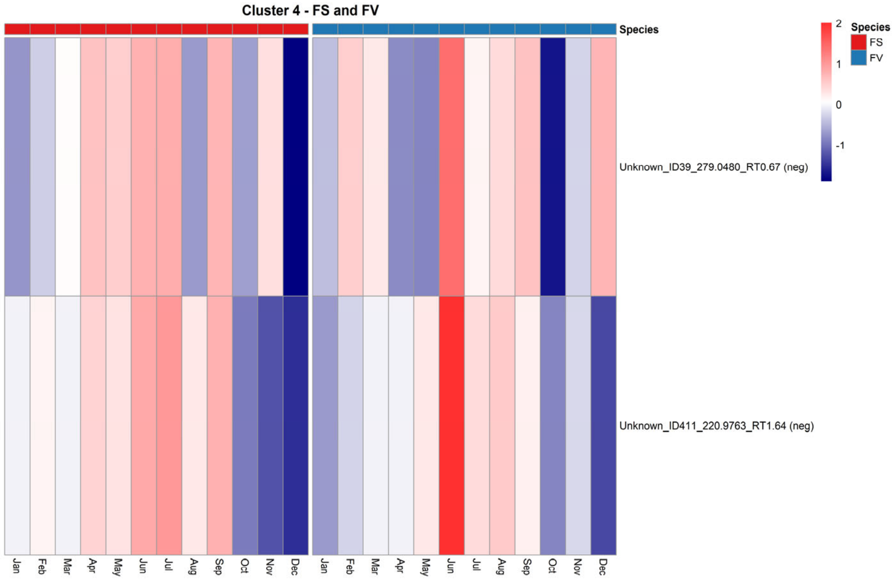

Supplementary Figure S3. Heatmap of standardized abundance (Z-score) for metabolites in Cluster 4 in *Fucus serratus* (FS, red bar) and *Fucus vesiculosus* (FV, blue bar). Each row represents a metabo-lite; each column represents a monthly sampling point from January to December.

Supplementary Table S1. Annotated features (positive and negative mode) from *Fucus serratus* and *Fucus vesiculosus* metabolic profiling, with LIMMA statistical descriptors, identification confidence and CANOPUS compound class predictions.

Nr	ID-m/z-rt 1	Name	Adduct	Molecular Formula	LIMMA (FC)2	LIMMA (P-val Adj)3	ID Level4	CSI:Finger ID Confidence	CANOPUS - Compound Class Prediction (Chemical category)	CANOPUS - Compound Class Prediction (Ancestors)5
				positive						
1	ID8_135.0474_rt0.67	2-Ethylsulfanylpropanoate (pos)	[M + H]+	C5H10O2S	-0.61	0.003	2	0.457	Fatty acids and conjugates	OC -> LLLM -> Fatty Acyls
2	ID14_206.0457_rt0.67	2-(3-Hydroxypropylamino)ethanesulfonic acid (pos)	[M + Na]+	C5H13NO4S	-1.23	<0.0001	2	0.579	Alkanesulfonic acids	OC -> OAD -> Organic sulfonic acids and derivatives -> Organosulfonic acids and derivatives -> Alkanesulfonic acids and derivatives
3	ID17_148.0609_rt0.67	Glutamic acid (pos)	[M + H]+	C5H9NO4	0.66	<0.0001	1	0.961	Alpha amino acids	OC -> OAD -> CAD -> AAPA -> Amino acids and derivatives
4	ID23_389.1773_rt0.68	Ala-Ala-Asn-Asn (pos)	[M + H]+	C14H24N6O7	ns	ns	3	0.182	Peptides	OC -> OAD -> CAD -> AAPA -> Peptides
5	ID28_275.1347_rt0.68	Gln-Gln (pos)	[M + H]+	C10H18N4O5	ns	ns	2	0.981	Peptides	OC -> OAD -> CAD -> AAPA -> Peptides
6	ID34_183.0863_rt0.68	Mannitol (pos)	[M + H]+	C6H14O6	ns	ns	2	0.833	Monosaccharides	OC -> OOC -> Organooxygen compounds -> Carbohydrates and carbohydrate conjugates
7	ID53_236.1492_rt0.70	4-aminobutyl alpha-L-fucopyranoside (pos)	[M + H]+	C10H21NO5	-0.51	<0.0001	3	0.052	Acyl carnitines	OC -> LLLM -> Fatty Acyls -> Fatty acid esters
8	ID54_146.1176_rt0.71	3-Dehydroxycarnitine (pos)	[M + H]+	C7H15NO2	ns	ns	3	0.957	Acyl carnitines	OC -> LLLM -> Fatty Acyls -> Fatty acids and conjugates
9	ID61_403.1931_rt0.72	Gln-Gln-Gln (pos)	[M + H]+	C15H26N6O7	1.32	0.0400	2	0.605	Peptides	OC -> OAD -> CAD -> AAPA -> Peptides
10	ID70_162.1125_rt0.72	Carnitine (pos)	[M + H]+	C7H15NO3	2.67	<0.0001	1	0.257	Acyl carnitines	OC -> LLLM -> Fatty Acyls -> Fatty acid esters
11	ID76_190.1074_rt0.75	Hydroxyoctadecadienylcarnitine (pos)	[M + H]+	C8H15NO4	-1.32	<0.0001	2	0.911	Acyl carnitines	OC -> LLLM -> Fatty Acyls -> Fatty acid esters
12	ID84_144.1019_rt0.75	Proline betaine (pos)	[M + H]+	C7H13NO2	ns	ns	1	0.81	Alpha amino acids	OC -> OAD -> CAD -> AAPA -> Amino acids and derivatives
13	ID116_162.0760_rt1.06	Glutamic acid gamma-methyl ester (pos)	[M + H]+	C6H11NO4	ns	ns	2	0.878	Alpha amino acids esters	OC -> OAD -> CAD -> AAPA -> Amino acids and derivatives

14	ID121_136.0616_rt1.08	Adenine (pos)	[M + H]+	C5H5N5	ns	ns	1	0.947	6-aminopurines	OC -> Organoheterocyclic compounds -> Imidazopyrimidines -> Purines and purine derivatives
15	ID124_346.1716_rt1.08	Gln-Gln-Ala (pos)	[M + H]+	C13H23N5O6	ns	ns	2	0.418	Peptides	OC -> OAD -> CAD -> AAPA -> Peptides
16	ID138_348.0699_rt1.09	Adenosine monophosphate (pos)	[M + H]+	C10H14N5O7P	0.89	<0.0001	1	0.854	Purine ribonucleoside monophosphates	OC -> Nucleosides, nucleotides, and analogues -> Purine nucleotides -> Purine ribonucleotides
17	ID163_258.1081_rt1.20	Pyroglutamylglutamine (pos)	[M + H]+	C10H15N3O5	ns	ns	2	0.982	Peptides	OC -> OAD -> CAD -> AAPA -> Peptides
18	ID174_193.0342_rt1.22	Citrate (pos)	[M + H]+	C6H8O7	ns	ns	1	0.904	Alpha hydroxy acids and derivatives	OC -> OAD -> Hydroxy acids and derivatives
19	ID175_210.0607_rt1.22	[M + H3N + H]+ Citrate (pos)	[M + H3N + H]+	C6H8O7	ns	ns	2	0.91	Alpha hydroxy acids and derivatives	OC -> OAD -> Hydroxy acids and derivatives
20	ID199_386.1664_rt1.49	Pyr-Gln-Gln (pos)	[M + H]+	C15H23N5O7	1.3	0.0206	2	0.484	Peptides	OC -> OAD -> CAD -> AAPA -> Peptides
21	ID206_246.1445_rt1.57	Glutaminylvaline (pos)	[M + H]+	C10H19N3O4	ns	ns	2	0.996	Peptides	OC -> OAD -> CAD -> AAPA -> Peptides
22	ID208_182.0811_rt1.59	Tyrosine (pos)	[M + H]+	C9H11NO3	0.83	0.0003	1	0.975	Alpha amino acids	OC -> OAD -> CAD -> AAPA -> Amino acids and derivatives
23	ID221_132.1018_rt1.72	Isoleucine (pos)	[M + H]+	C6H13NO2	1.32	<0.0001	1	0.794	Alpha amino acids	OC -> OAD -> CAD -> AAPA -> Amino acids and derivatives
24	ID226_268.1038_rt1.87	Adenosine (pos)	[M + H]+	C10H13N5O4	-1.27	<0.0001	1	0.976	Purine nucleosides	OC -> Nucleosides, nucleotides, and analogues
25	ID248_513.0657_rt2.21	Hydrolyzable tannin	[M + Na]+	C22H18O13	-5.08	<0.0001	3	0.689	Hydrolyzable tannins	OC -> Phenylpropanoids and polyketides -> Tannins -> Hydrolyzable tannins
26	ID257_207.0498_rt2.36	Methyl citrate (pos)	[M + H]+	C7H10O7	-0.74	0.0098	2	0.837	Fatty acid methyl esters	OC -> LLLM -> Fatty Acyls -> Fatty acid esters
27	ID265_389.0497_rt2.47	5,6,7,8,2',3',5'-heptahydroxy-4'-methoxyflavanone (pos)	[M + Na]+	C16H14O10	-1.24	<0.0001	2	0.162	Flavonoids	OC -> Phenylpropanoids and polyketides -> Flavonoids -> O-methylated flavonoids -> 4'-O-methylated flavonoids

28	ID273_166.0862_rt2.53	Phenylalanine (pos)	[M + H]+	C9H11NO2	0.67	<0.0001	1	0.994	Alpha amino acids	OC -> OAD -> CAD -> AAPA -> Amino acids and derivatives
29	ID307_513.0659_rt3.09	Hydrolyzable tannin	[M + Na]+	C22H18O13	-2.13	<0.0001	3	0.127	Hydrolyzable tannins	OC -> Phenylpropanoids and polyketides -> Tannins -> Hydrolyzable tannins
30	ID308_388.2185_rt3.01	Gln-Leu-Gln (pos)	[M + H]+	C16H29N5O6	ns	ns	2	0.551	PolyPeptides	OC -> OAD -> CAD -> AAPA -> Peptides
31	ID319_205.0971_rt3.45	Tryptophan (pos)	[M + H]+	C11H12N2O2	0.67	<0.0001	1	0.914	Alpha amino acids	OC -> OAD -> CAD -> AAPA -> Amino acids and derivatives -> Alpha amino acids and derivatives
32	ID321_422.2029_rt3.51	Gln-Gln-Phe (pos)	[M + H]+	C19H27N5O6	ns	ns	2	0.462	Peptides	OC -> OAD -> CAD -> AAPA -> Peptides
33	ID325_623.1026_rt3.54	Isoorientin 2''-O-Gallate (pos)	[M + Na]+	C28H24O15	-6.4	<0.0001	2	0.199	Flavonoid-3-O-glycosides	OC -> Phenylpropanoids and polyketides -> Flavonoids -> Flavonoid glycosides -> Flavonoid O-glycosides
34	ID332_623.1025_rt3.73	Isoorientin 2''-O-Gallate isomer (pos)	[M + Na]+	C28H24O15	-5.84	<0.0001	2	0.368	Flavonoid-3-O-glycosides	OC -> Phenylpropanoids and polyketides -> Flavonoids -> Flavonoid glycosides -> Flavonoid O-glycosides
35	ID385_205.0795_rt5.71	1-(2-phenylethyl)-1H-imidazole-2-thiol (pos)	[M + H]+	C11H12N2S	ns	ns	2	0.224	Benzenoids	OC
36	ID389_197.1172_rt5.80	Loliolide (pos)	[M + H]+	C11H16O3	ns	ns	2	0.133	Butenolides	OC -> Organoheterocyclic compounds -> Dihydrofurans -> Furanones
37	ID444_381.2425_rt8.88	Niuhinone C (pos)	[M + Na]+	C23H34O3	ns	ns	2	0.209	Acyclic diterpenoids	OC -> LLLM-> Prenol lipids-> Diterpenoids-> Acyclic diterpenoids
38	ID453_263.1643_rt9.33	O-Methylperezone (pos)	[M + H]+	C16H22O3	ns	ns	2	0.136	Sesquiterpenoids	OC -> LLLM-> Prenol lipids-> Sesquiterpenoids
					negative mode					
1	ID1_146.0460_rt0.66	Glutamic acid (neg)	[M - H]-	C5H9NO4	1.37	<0.0001	1	0.949	Alpha amino acids	OC -> OAD -> CAD -> AAPA -> Amino acids and derivatives

2	ID9_182.0495_rt0.67	Choline sulfate (neg)	[M - H]-	C5H13NO4S	-0.64	<0.0001	2	0.026	Alkanesulfonic acids	OC -> OAD -> Organic sulfonic acids and derivatives -> Organosulfonic acids and derivatives -> Alkanesulfonic acids and derivatives
3	ID12_219.0461_rt0.67	2-Deoxy-2-fluoro-D-glucitol (neg)	[M + Cl]-	C6H13FO5	ns	ns	2	0.501	Monosaccharides	OC -> OOC -> Organooxygen compounds -> Carbohydrates and carbohydrate conjugates
4	ID24_217.0488_rt0.67	[M + Cl]- Mannitol (neg)	[M + Cl]-	C6H14O6	ns	ns	2	0.426	Monosaccharides	OC -> OOC -> Organooxygen compounds -> Carbohydrates and carbohydrate conjugates
5	ID29_260.9983_rt0.67	2-(Sulfamoylamino)pentanedioic acid (neg)	[M + Cl]-	C5H10N2O6S	ns	ns	2	0.043	Alpha amino acids	OC -> OAD -> CAD -> AAPA -> Amino acids and derivatives
6	ID31_387.1633_rt0.67	Gln-Gln-Asn (neg)	[M - H]-	C14H24N6O7	ns	ns	2	0.079	Alpha amino acids	OC -> OAD -> CAD -> AAPA -> Amino acids and derivatives
7	ID36_385.1164_rt0.67	1-O-Sinapoylglucose (neg)	[M - H]-	C17H22O10	ns	ns	2	0.38	Coumaric acids and derivatives	OC -> Phenylpropanoids and polyketides -> Cinnamic acids and derivatives -> Hydroxycinnamic acids and derivatives
8	ID58_399.0958_rt0.68	Sinapinic Acid-O-Glucuronide Isomer (neg)	[M - H]-	C17H20O11	ns	ns	2	0.383	Glucuronic acid derivatives	OC -> OOC -> Organooxygen compounds -> Carbohydrates and carbohydrate conjugates -> Sugar acids and derivatives
9	ID63_155.0021_rt0.68	3-Sulfopropanediol (neg)	[M - H]-	C3H8O5S	ns	ns	2	0.611	Alkanesulfonic acids	OC -> OAD -> Organic sulfonic acids and derivatives -> Organosulfonic acids and derivatives -> Alkanesulfonic acids and derivatives
10	ID104_379.1017_rt0.71	[M + Cl]- Maltitol (neg)	[M + Cl]-	C12H24O11	0.98	0.0360	2	0.172	Alkyl glycosides	OC -> LLLM -> Fatty Acyls -> Fatty acyl glycosides
11	ID112_343.1253_rt0.71	Maltitol (neg)	[M - H]-	C12H24O11	1.08	0.0182	2	0.317	Alkyl glycosides	OC -> LLLM -> Fatty Acyls -> Fatty acyl glycosides
12	ID129_402.1820_rt0.72	Trp-Ala-Gln (neg)	[M - H]-	C19H25N5O5	ns	ns	2	0.234	OligoPeptides	OC -> OAD -> CAD -> AAPA -> Peptides

13	ID134_181.0720_rt0.71	Mannitol (neg)	[M - H]-	C6H14O6	ns	ns	2	0.537	Monosaccharides	OC -> OOC -> Organooxygen compounds -> Carbohydrates and carbohydrate conjugates
14	ID136_499.1492_rt0.73	Glu-Tyr-Ser-Cys (neg)	[M - H]-	C20H28N4O9S	ns	ns	2	0.105	OligoPeptides	OC -> OAD -> CAD -> AAPA -> Peptides
15	ID228_110.9759_rt0.76	Hydroxymethanesulfonic acid (neg)	[M - H]-	CH4O4S	ns	ns	2	0.325	Alkanesulfonic acids	OC -> OAD -> Organic sulfonic acids and derivatives -> Organosulfonic acids and derivatives -> Alkanesulfonic acids and derivatives
16	ID309_111.0087_rt1.12	2-Furoic Acid (neg)	[M - H]-	C5H4O3	-1.31	<0.0001	2	0.844	Furoic acid	OC -> Organoheterocyclic compounds -> Furans -> Furoic acid and derivatives
17	ID316_173.0093_rt1.09	Isocitric acid lactone (neg)	[M - H]-	C6H6O6	-5.11	<0.0001	3	0.56	Tricarboxylic acids and derivatives	OC -> OAD -> CAD
18	ID321_370.1366_rt1.09	Ser-His-Glu (neg)	[M - H]-	C14H21N5O7	ns	ns	2	0.105	OligoPeptides	OC -> OAD -> CAD -> AAPA -> Peptides
19	ID357_191.0200_rt1.22	Citrate (neg)	[M - H]-	C6H8O7	ns	ns	1	0.783	Tricarboxylic acids and derivatives	OC -> OAD -> CAD
20	ID382_498.1953_rt1.33	Ac-Gly-Gly-Asp-Pro-Gly-Gly (neg)	[M - H]-	C19H29N7O9	ns	ns	2	0.956	OligoPeptides	OC -> OAD -> CAD -> AAPA -> Peptides
21	ID390_384.1523_rt1.49	Pyr-Gln-Gln (neg)	[M - H]-	C15H23N5O7	1.28	0.0224	2	0.916	OligoPeptides	OC -> OAD -> CAD -> AAPA -> Peptides
22	ID399_173.0093_rt1.53	Aconitic Acid (neg)	[M - H]-	C6H6O6	-2.55	<0.0001	1	0.893	Tricarboxylic acids and derivatives	OC -> OAD -> CAD
23	ID404_243.0620_rt1.59	Uridine (neg)	[M - H]-	C9H12N2O6	ns	ns	1	0.886	Pyrimidine nucleosides	OC -> Nucleosides, nucleotides, and analogues
24	ID409_611.1448_rt1.62	GSSG (Oxidized glutathione) (neg)	[M - H]-	C20H32N6O12S2	0.74	0.0013	2	0.563	Gamma-glutamyl Peptides	OC -> OAD -> CAD -> AAPA -> Peptides
25	ID484_373.0565_rt2.26	Trifucol (neg)	[M - H]-	C18H14O9	-4.7	<0.0001	2	0.055	Epigallocatechins	OC -> Phenylpropanoids and polyketides -> Flavonoids -> Flavans -> Flavan-3-ols -> Catechins
26	ID495_205.0354_rt2.35	Methyl citrate (neg)	[M - H]-	C7H10O7	-1.01	0.0053	2	0.669	Tricarboxylic acids and derivatives	OC -> OAD -> CAD

27	ID497_111.0085_rt2.36	2-Furoic Acid (isomer) (neg)	[M - H]-	C5H4O3	-0.86	0.0031	2	0.837	Furoic acid	OC -> Organoheterocyclic compounds -> Furans -> Furoic acid and derivatives
28	ID505_387.0357_rt2.41	Quercetin pyruvate (neg)	[M - H]-	C18H12O10	-3.04	<0.0001	2	0.567	Flavonols	OC -> Phenylpropanoids and polyketides -> Flavonoids -> Flavones
29	ID528_246.9919_rt2.46	2-[4-Hydroxy-3-(sulfooxy)phenyl]acetic acid (neg)	[M - H]-	C8H8O7S	ns	ns	2	0.269	Phenylsulphates	OC -> OAD -> Organic sulfuric acids and derivatives -> Arylsulfates
30	ID538_167.0350_rt2.70	Homogentisic acid (neg)	[M - H]-	C8H8O4	ns	ns	2	0.795	2(hydroxyphenyl)acetic acids	OC -> Benzenoids -> Benzene and substituted derivatives -> Phenylacetic acids
31	ID565_497.0728_rt2.89	Fucodiphloroethol G (neg)	[M - H]-	C24H18O12	-6.34	<0.0001	2	0.012	Catechin gallates	OC -> Phenylpropanoids and polyketides -> Flavonoids -> Flavans -> Flavan-3-ols -> Catechins
32	ID582_497.0727_rt2.99	Fucodiphloroethol G (isomer) (neg)	[M - H]-	C24H18O12	-6.71	<0.0001	2	0.084	Catechin gallates	OC -> Phenylpropanoids and polyketides -> Flavonoids -> Flavans -> Flavan-3-ols -> Catechins
33	ID594_261.0075_rt3.19	Dihydrocaffeic acid 3-sulfate (neg)	[M - H]-	C9H10O7S	4.15	<0.0001	2	0.402	Phenylsulphates	OC -> OAD -> Organic sulfuric acids and derivatives -> Arylsulfates
34	ID603_511.0519_rt3.31	Diphlorethohydroxycarmalol (neg)	[M - H]-	C24H16O13	-4.19	<0.0001	2	0.029	3'-hydroxyflavonoids	OC -> Phenylpropanoids and polyketides -> Flavonoids -> Hydroxyflavonoids
35	ID626_230.9970_rt3.61	4-Hydroxyphenylacetic acid sulfate (neg)	[M - H]-	C8H8O6S	1.86	<0.0001	2	0.411	Phenylsulphates	OC -> OAD -> Organic sulfuric acids and derivatives -> Arylsulfates
36	ID646_261.0074_rt3.78	Homovanillic Acid Sulfate (neg)	[M - H]-	C9H10O7S	ns	ns	2	0.733	Phenylsulphates	OC -> OAD -> Organic sulfuric acids and derivatives -> Arylsulfates
37	ID700_275.0230_rt4.41	Dihydroferulic acid 4-O-sulfate (neg)	[M - H]-	C10H12O7S	4.04	<0.0001	2	0.507	Phenylsulphates	OC -> OAD -> Organic sulfuric acids and derivatives -> Arylsulfates

38	ID938_527.2530_rt14.59	Sulfoquinovosyl monoacyl-glyceride alpha (neg)	[M - H]-	C23H44O11S	ns	ns	3	0.418	Glycosylglycerols	OC -> LLLM -> Glycerolipids

*[1]ID, identification number from mzmine analysis pipeline to ensure traceability of features, m/z – mass-to-charge ratio; rt – retention time; 2FC – fold change results after the LIMMA analysis, positive FC values (>0.5) indicate higher intensities in the FS, negative FC values (<-0.5) indicate higher intensities in FV; 3P-values from LIMMA analysis comparison, adjusted for multiple comparison using Benjamini-Hochberg correction; 4Compound identification level according to Sumner et al (2014); 5Abbreviations of compound classes: Organic compounds (OC), Lipid and lipid-like molecules (LLLM), Organic acids and derivatives (OAD), Carboxylic acids and analogues (CAD), Amino acids, peptides and analogues (AAPA), Organic oxygen compounds (OOC)
